# Induction of autophagy in rats upon overexpression of wild-type and mutant optineurin gene

**DOI:** 10.1186/s12860-015-0060-x

**Published:** 2015-05-06

**Authors:** Hongyu Ying, Sanja Turturro, Tara Nguyen, Xiang Shen, Ruth Zelkha, Elaine C Johnson, John C Morrison, Beatrice YJT Yue

**Affiliations:** Department of Ophthalmology and Visual Sciences, University of Illinois at Chicago, College of Medicine, Chicago, IL USA; Department of Ophthalmology, Casey Eye Institute, Oregon Health and Science University, Portland, OR USA

**Keywords:** Optineurin, Glaucoma, Amyotrophic lateral sclerosis, Ubiquitin-proteasome pathway (UPP), Autophagy, Adeno-associated type 2 viral (AAV2) vectors, E50K mutation, Rat

## Abstract

**Background:**

Optineurin is a gene associated with normal tension glaucoma and amyotrophic lateral sclerosis. It has been reported previously that in cultured RGC5 cells, the turnover of endogenous optineurin involves mainly the ubiquitin-proteasome pathway (UPP). When optineurin is upregulated or mutated, the UPP function is compromised as evidenced by a decreased proteasome β5 subunit (PSMB5) level and autophagy is induced for clearance of the optineurin protein.

**Results:**

Adeno-associated type 2 viral (AAV2) vectors for green fluorescence protein (GFP) only, GFP-tagged wild-type and Glu50Lys (E50K) mutated optineurin were intravitreally injected into rats for expression in retinal ganglion cells (RGCs). Following intravitreal injections, eyes that received optineurin vectors exhibited retinal thinning, as well as RGC and axonal loss compared to GFP controls. By immunostaining and Western blotting, the level of PSMB5 and autophagic substrate degradation marker p62 was reduced, and the level of autophagic marker microtubule associated protein 1 light chain 3 (LC3) was enhanced. The UPP impairment and autophagy induction evidently occurred *in vivo* as *in vitro*. The optineurin level, RGC and axonal counts, and apoptosis in AAV2-E50K-GFP-injected rat eyes were averted to closer to normal limits after treatment with rapamycin, an autophagic enhancer.

**Conclusions:**

The UPP function was reduced and autophagy was induced when wild-type and E50K optineurin was overexpressed in rat eyes. This study validates the *in vitro* findings, confirming that UPP impairment and autophagy induction also occur *in vivo*. In addition, rapamycin is demonstrated to clear the accumulated mutant optineurin. This agent may potentially be useful for rescuing of the adverse optineurin phenotypes *in vivo*.

## Background

Glaucoma is one of the leading causes of irreversible bilateral blindness worldwide. Optineurin or the “optic neuropathy inducing” gene was discovered in 2002 [1] to be a candidate gene of primary open-angle glaucoma (POAG), the most common form of glaucoma. POAG, characterized by degeneration of retinal ganglion cells (RGCs) and progressive axonal and visual field loss, is age-related and frequently associated with increased intraocular pressure (IOP) [2]. It is genetically heterogeneous, caused by several susceptibility genes [3-5] as well as environmental factors [5]. Optineurin was found to be linked in particular to normal tension glaucoma (NTG) [3,6], a subtype of POAG. Optineurin mutations were noted to vary with ethnic background [7]. The Glu50Lys (E50K) mutation, found in Caucasian and Hispanic populations, seems to be associated with a more progressive and severe disease in NTG patients [8].

More recently, mutations in optineurin have also been reported to associate with amyotrophic lateral sclerosis (ALS) [9,10]. In addition, optineurin was identified as one of the genetic risk factors for Paget’s disease of bone [11,12].

The human optineurin gene codes for a 577-amino acid protein [1]. The optineurin protein consists of a NF-κB essential molecule (NEMO)-like domain, leucine zipper motif, multiple coiled-coil motifs, an ubiquitin binding domain, a microtubule associated protein 1 light chain 3 (LC3)-interacting motif, and a carboxyl (C)-terminal zinc finger [13]. Optineurin is a cytosolic protein that is not secreted [14]. It is expressed in many non-ocular tissues such as the brain and the heart as well as in ocular tissues including the retina and the trabecular meshwork [13,15].

Optineurin has been shown to be a negative regulator of the NF-κB pathway [16-20], an autophagy receptor [21-23], as well as a player in antiviral immune response [24] and mitotic progression [25,26]. This protein is moreover demonstrated to interact and form complexes with proteins including Rab8, huntingtin, myosin VI, and transferrin receptor, and such interactions may be the basis why optineurin is also involved in regulation of protein trafficking [27-31].

It has been previously reported that in cultured RGC5 cells, optineurin is ubiquitinated and the turnover of endogenous optineurin involves mainly the ubiquitin proteasome pathway (UPP) in normal homeostatic situation [32]. When optineurin is upregulated or mutated, the level of proteasome β5 subunit (PSMB5), an indicator of proteasome activity [33,34], is reduced, signifying a compromised UPP function [32]. The level of the lipidated form of LC3, LC3-II, is elevated, indicating that autophagosome formation/autophagy is increased [35]. Additionally, the level of p62, a maker for autophagy substrate degradation [35-37], was concurrently found declined in E50K optineurin-transfected RGC5 cells [Ying and Yue, unpublished observations submitted for publication], suggesting an activation of both autophagy and autophagic flux.

To validate the *in vitro* results and substantiate their relevance, especially in light of the recent finding that the originally thought rat retinal ganglion RGC5 cell line is in fact 661 W [38], a mouse SV-40 T antigen transformed photoreceptor cell line, the present *in vivo* investigation was undertaken. Adeno-associated type 2 viral (AAV2) vectors for green fluorescence protein (GFP), GFP-tagged wild-type and E50K optineurin were intravitreally injected into rats for expression in RGCs. The purpose was to determine whether impairment of UPP and/or induction of autophagy would take place *in vivo* as *in vitro*. Additionally, the study examined whether the accumulated mutant optineurin could be efficiently cleared by rescuing strategies such as the use of autophagic enhancer rapamycin.

## Results

### Normal IOP in rAAV transduced eyes

**Figure 1:**
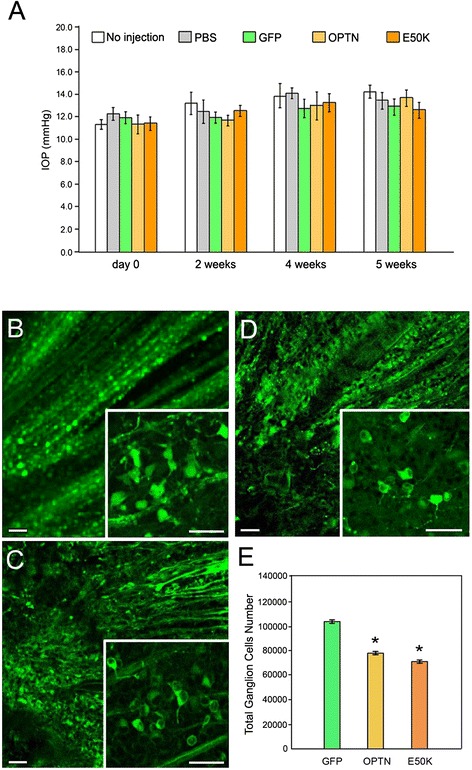
IOP readings and retinal ganglion cell counts in AAV2 viral vector injected rat eyes. **A**. IOP measurements. One eye of the rats was intravitreally injected with one of the AAV2 viral vectors (GFP, green bars; OPTN-GFP or OPTN, yellow bars; E50K-GFP or E50K, orange bars), and the contralateral eye was either non-injected (white bars) or injected with PBS (gray bars) as negative controls. The IOP was measured before injection (referred to as day 0), or 2, 4, and 5 weeks post-injection. Results are presented as mean ± SEM (n =15). No IOP difference was seen between AAV2 injection and control groups. **B**-**D**. Retinal whole mounts. Rat eyes were injected intravitreally with one of the AAV2 viral vectors (GFP, **B**; OPTN-GFP or OPTN, **C**; and E50K-GFP or E50K, **D**). Five weeks post-injection, the retinal whole mounts were visualized by fluorescence microscopy. Efficient transduction in cells in the retinal ganglion layer was observed. The GFP expression was robust with clearly visible retinal ganglion axons. Insets show other areas at a higher magnification. Scale bars, 20 μm. **E**. Bar graphs depict total retinal ganglion cell counts per retina. Results are presented as mean ± SEM (n = 6). *, P < 0.0062 compared with GFP controls.

To introduce gene expression in the RGC layer in the retina, AAV2-GFP-, AAV2-optineurin (OPTN)-GFP-, and AAV2-E50K-GFP-vectors were injected intravitreally into the left eyes of Brown Norway rats. The IOP was monitored at day 0 (before injection), and weeks 2, 4 and 5 post-injection using rebound tonometer TonoLab in awake rats. No significant changes were observed in any of the injected eyes compared with PBS-injected or non-injected controls (Figure 1A). The IOP during the course of experiments was in general 11–14 mmHg in all of the rat eyes as expected.

### Transgene expression

The efficiency of transgene expression was assessed in retinal whole mounts. After enucleation of the globe, the anterior segment was removed and the posterior segment was fixed briefly. The retina was then isolated, divided into quadrants and flat mounted onto a microscopic slide with the RGC side up. The GFP-positive cells were visualized and the number of GFP positive cells in digital images of 20 randomly sampled 20x whole mount fields was determined. Transduction of AAV2-GFP (Figure 1B), AAV2-OPTN-GFP (Figure 1C) and AAV2-E50K-GFP (Figure 1D) was found highly and equally efficient in cells of the RGC layer. The total number of GFP-positive cells was 105718 ± 9047 per retina in the AAV2-GFP treated control eyes. The cell count was significantly (P < 0.0062) reduced to 79838 ± 9368 and 71874 ± 9865/retina respectively in AAV2-OPTN-GFP- and AAV2-E50K-GFP-injected eyes (Figure 1E).

### Degeneration of optic nerve axons in optineurin transduced eyes

**Figure 2:**
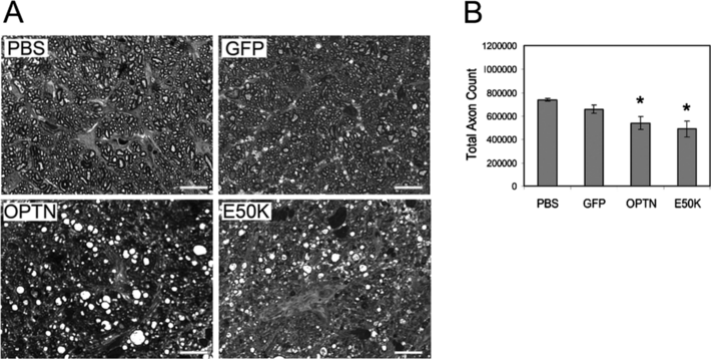
Optic nerve axons in AAV2 viral vector injected rat eyes. **A**. High-power view of representative optic nerve axon cross sections of rats 5 weeks following injection of PBS, or one of the viral vectors (GFP, OPTN-GFP or OPTN, E50K-GFP or E50K). PBS injection was used as negative control. Magnification, x100. Scale bar, 20 μm. The axons in PBS- and GFP vector-injected eyes showed normal morphology while densely stained degenerating axons as well as axonal swellings were seen in wild-type OPTN- and E50K-GFP-vector injected eyes. **B**. Total axon counts. The value (mean ± SEM) in wild-type OPTN-GFP injected eyes was significantly (P < 0.0042, asterisks, n =15) lower than that in PBS and GFP injected control eyes. A greater decrease of total axon count was found in E50K-GFP injected eyes.

To examine the optic nerve axons, optic nerves in the enucleated eyes were fixed and processed. Semi-thin sections prepared were stained with toluidine blue. Subsequent analysis of the optic nerve axons using high-power light microscopy demonstrated normal axonal features in AAV2-GFP-treated and PBS-injected control eyes. By contrast, densely stained degenerating axons as well as axonal swellings were observed, interspersed with normal axons, in eyes administered with optineurin and E50K viral constructs (Figure 2A). Quantification of axonal counts indicated a significantly (P < 0.0042) decreased axon count in AAV2-OPTN-GFP-injected (540323 ± 54213) eyes compared to PBS (739059 ± 12434) and GFP (658955 ± 35326) controls. A greater degree of axon decrease was seen in E50K-expressing (489696 ± 69261) specimens (Figure 2B).

### Proteasomal compromise and autophagy induction in optineurin transduced eyes

**Figure 3:**
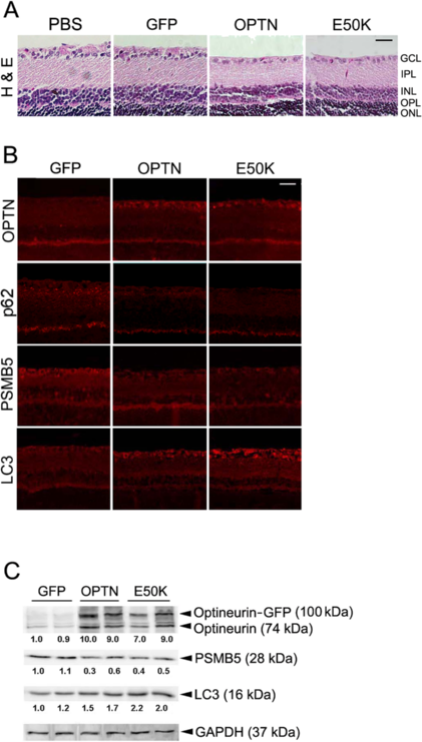
Histology and levels of optineurin, p62, PSMB5 and LC3 in the retina of viral vector injected rat eyes. **A**. Representative retinal sections collected following injection of PBS, and GFP-, wild-type OPTN (OPTN)- and E50K-OPTN (E50K)-GFP vectors were stained with hematoxylin and eosin (H & E). GCL, ganglion cell layer; IPL, inner plexiform layer; INL, inner nuclear layer; OPL, outer plexiform layer; and ONL, outer nuclear layer. The retinal ganglion cell number was decreased and the INL thickness was reduced in OPTN- and E50K-injected specimens compared to PBS and GFP controls. Scale bar, 25 μm. **B**. Retinal sections from rat eyes were stained in parallel with polyclonal anti-optineurin, p62, PSMB5, or LC3 (all in red). An enhanced optineurin (OPTN) and LC3 staining, and a declined PSMB5 and p62 staining were observed in the retinal ganglion layer of OPTN and E50K-injected eyes compared to controls. Scale bar, 25 μm. **C**. Western blotting for optineurin, PSMB5, LC3, and GAPDH in retinal extracts from GFP-, OPTN-GFP (OPTN)-, and E50K-OPTN-GFP (E50K)-injected eyes. The LC3-I bands were very faint and only those of LC3-II were shown. The level of optineurin overexpression was estimated by comparing the intensities of both (optineurin-GFP + optineurin) bands from optineurin treatment groups to those of the endogenous optineurin band from GFP groups (all were normalized against GAPDH). The optineurin/GAPDH ratios (n = 3) for the GFP, OPTN, and E50K groups were, respectively, 1.0 ± 0.2, 9.5 ± 0.3, and 8.0 ± 0.5. For PSMB5/GAPDH, the corresponding values (relative to the first GFP sample) were 1.1 ± 0.1, 0.5 ± 0.1, and 0.5 ± 0.1; and for LC3/GAPDH, they were 1.0 ± 0.1, 1.6 ± 0.2, and 2.1 ± 0.2. All OPTN and E50K results were significantly (P < 0.0015) different from those of GFP controls.

Retinal cross sections were prepared for hematoxylin and eosin (H and E) staining to assess retinal changes in optineurin transduced rat eyes. It was noted that 5 weeks following AAV2-OPTN-GFP or AAV2-E50K-GFP injection, the retina showed a reduced number of ganglion cells and thinning of the inner nuclear layer compared to PBS (Figure 3A)- or AAV2-GFP (Figure 3A)- injected as well as non-injected (data not shown) controls.

The expression level of optineurin, p62, PSMB5, and LC3 in the RGC layer was investigated in retinal cross sections by immunostaining. As anticipated, retinal sections from wild-type and E50K optineurin transduced eyes yielded a stronger optineurin staining than the controls (Figure 3B, top panel). They in addition displayed a fainter staining of p62 (Figure 3B, second panel) and PSMB5 (Figure 3B, third panel), and a stronger staining of LC3 (Figure 3B, bottom panel) in RGCs. Western blot analyses further revealed that the PSMB5 protein level was decreased by approximately 50% while the optineurin level (optineurin and GFP-tagged optineurin) was increased by 8–9 fold upon transduction of wild-type and E50K optineurin in rat eyes (Figure 3C).

LC3 exists in two forms. LC3-I (18 kDa) is cytosolic and LC3-II (16 kDa) is the lipidated form (conjugated to phosphatidylethanolamine) that inserts into the membrane. The amount of LC3-II is correlated with the extent of autophagosome formation and increasing levels of LC3-II on immunoblots have been used to document induction of autophagy [35]. In rats, the level of LC3-II protein in the retina was found increased by approximately 1.6-2.0 fold by Western blotting in AAV2-optineurin wild-type and E50K-treated eyes (Figure 3C). p62, an ubiquitin-binding scaffold protein [36], is itself degraded by autophagy and serves as a maker for autophagy substrate degradation [35,36]. A decreased p62 level has been correlated with activation of autophagy and autophagic flux and an increased p62 level correlates with autophagic suppression [37]. The observed weaker p62 staining corroborated an induced autophagy and enhanced autophagic flux in wild-type and E50K optineurin transduced eyes.

### Apoptosis level was elevated in RGCs in optineurin transduced eyes

**Figure 4:**
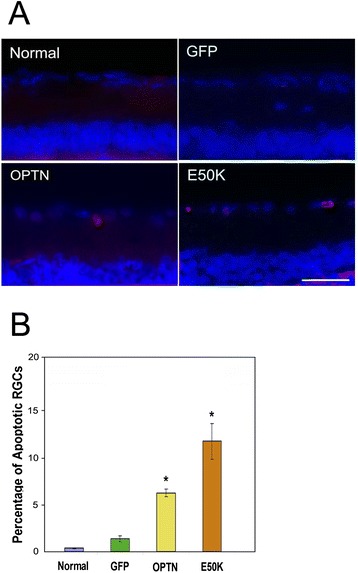
Apoptosis of cells in the retinal ganglion layer of rat eyes. **A**. Apoptotic cells in the retinal ganglion layer in eyes non-injected (Normal), and those 5 weeks post-injected with GFP, OPTN-GFP (OPTN), and E50K-GFP (E50K) viral vectors. A representative image from each group was shown. Retinal sections were examined by an ApopTag kit. Images (20 of 10× fields) were captured and cell counting was performed to determine the total number of retinal ganglion cells (RGCs) and the number of apoptotic ones (pink). Scale bar, 25 μm. **B**. Percentage of apoptotic RGCs was calculated. Results are shown in mean ± SEM (n = 10). The values for normal control, and GFP, OPTN, and E50K groups were, respectively, 0.3 ± 0.02, 1.4 ± 0.3, 6.2 ± 0.4, and 11.7 ± 1.9. *, P < 0.0005 compared with non-injected normal and GFP controls.

TdT-Mediated dUTP nick-end labeling (TUNEL) assay was carried out to assess apoptosis in the RGC layer. Retinal tissue sections from rats 5 weeks following the AAV2-OPTN-GFP or AAV2-E50K-GFP injection were found to exhibit a much higher percentage (6.2 ± 0.4 and 11.7 ± 1.9 respectively) of apoptotic RGCs than AAV2-GFP (1.4 ± 0.3) and non-injected (0.3 ± 0.02) controls (Figure 4).

### Proteasomal compromise and autophagy induction in rat eyes with chronic hypertension

**Figure 5:**
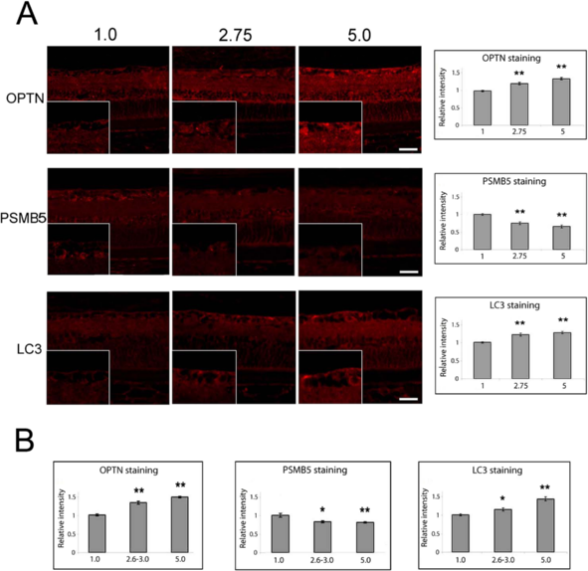
Immunostaining of optineurin (OPTN), PSMB5, and LC3 in the eyes of a rat chronic hypertension model. The optic nerve damage in the rats was graded from 1 (no injury) to 5 (active degeneration in the entire optic nerve area). **A**. Retinal cross sections of grades 1, 2.75, and 5 were immunostained. The grade 2.75 and 5 slides showed stronger OPTN and LC3, but weaker PSMB5, staining, compared with that of grading 1.0 (left panel). Higher magnification of staining was shown in insets. Scale bars, 25 μm. Images (20 of 10× fields) were captured and the intensity of each staining in retinal ganglion cells was quantified. Results (mean ± SEM, averaged from 40 cells total) are presented in the bar graph at the right panel. The staining intensity (relative to no injury control grade 1) for optineurin was 1.0 ± 0.04, 1.22 ± 0.04, and 1.36 ± 0.03, respectively, for grades 1, 2.75, and 5. The corresponding relative intensity for PSMB5 was 1.0 ± 0.03, 0.76 ± 0.04, and 0.66 ± 0.04; and that for LC3 was 1.0 ± 0.04, 1.20 ± 0.04 and 1.26 ± 0.03. **B**. Bar graphs depict the staining intensity of OPTN, PSMB5, and LC3 in retinal ganglion cells in another set of experiments. The retinal cross sections of grades 1.0, 2.58, 2.75, 3.0, and 5.0 were used. The relative staining intensity (mean ± SEM, averaged from 18–31 cells) for optineurin was 1.0 ± 0.02, 1.30 ± 0.03, and 1.43 ± 0.01, respectively, for grades 1, 2.6-3.0, and 5. The corresponding intensity for PSMB5 was 1.0 ± 0.04, 0.88 ± 0.02, and 0.86 ± 0.01; and that for LC3 was 1.0 ± 0.02, 1.13 ± 0.04, and 1.38 ± 0.05. **, P < 0.002; *, P < 0.032 compared to grade 1.

Hypertonic saline was injected into episcleral veins of Brown Norway rat to obstruct the aqueous outflow to achieve chronic IOP elevation [39,40]. The optic nerve damage in this rat model was graded from 1 (no injury) to 5 (active degeneration in the entire optic nerve area) [39]. In one set of experiment, sections with optic nerve damage graded as 1, 2.75 (degenerating axons and axonal swelling spread more than the focal area), and 5 were used. The sections were deparaffinized and stained in parallel for optineurin, PSMB5, and LC3. Analogous to wild-type and E50K optineurin transduced eyes, an enhanced optineurin, reduced PSMB5 and increased LC3 staining was observed in the RGC layer of sections from injury grades 2.75 and 5 compared to injury grade 1.0 (normal) in the rat ocular hypertension model (Figure 5A, left panel). Quantification analyses confirmed that the optineurin and LC3 levels in the RGC layer were higher, and the PSMB5 level was lower with advanced optic nerve injury. The alterations were more notable with increasing injury grades (Figure 5A, right panel). In another set of experiment, sections with optic nerve damage graded as 1, 2.6 to 3.0, and 5 were stained. An increased optineurin and LC3 staining and a decreased PSMB5 staining, were likewise discerned especially with grade 5 optic nerve damage (Figure 5B). These results suggested that an inhibition of proteasomal activity and induction of autophagy might also occur in hypertensive eyes in the rat experimental model.

### Effects of rapamycin

**Figure 6:**
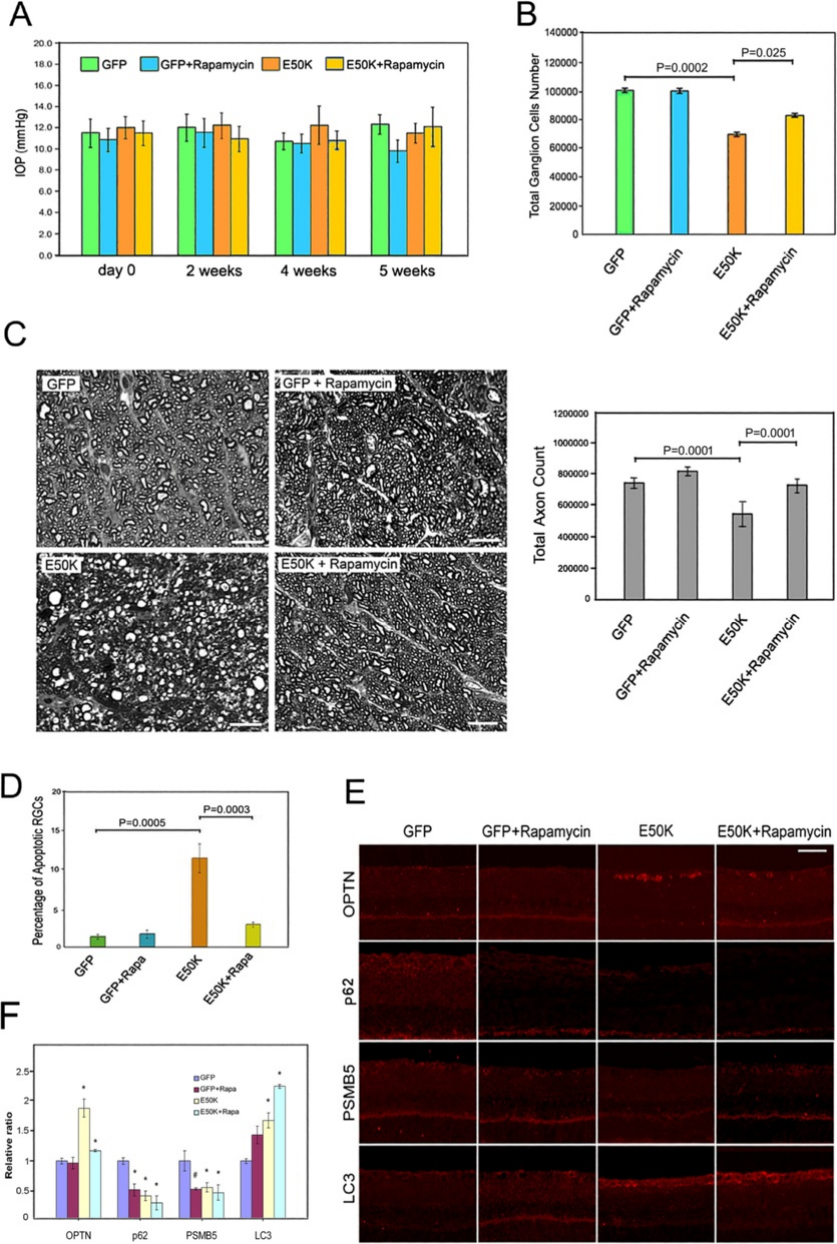
Effects of rapamycin. **A**. IOP values in rat eyes treated or untreated with rapamycin. Two sets of rats were injected with GFP (green and blue bars) or E50K-GFP (orange and yellow bars) viral vector. One set (blue and yellow bars) received intraperitoneal injection of rapamycin. The other set did not receive rapamycin treatment as controls. No change in IOP was observed without or with rapamycin treatment. **B**. Bar graphs depict total retinal ganglion cell counts per retina in retinal whole mounts from GFP or E50K-GFP viral vector injected eyes. One set of the animals received rapamycin treatment. Results are presented as mean ± SEM (n = 4 for GFP and n = 10 for E50K groups). **C**. Representative high-power view (100x) of optic nerve axon cross sections from eyes collected 5 weeks following intravitreal injection of GFP or E50K-GFP viral vector without or with treatment of rapamycin (left panel). Scale bar, 20 μm. Total axon counts in different groups are shown in the bar graph (right panel). **D**. Percentage of apoptotic cells in the retinal ganglion cell layer (left panel) in sections from GFP or E50K-GFP viral vector injected eyes without or with rapamycin (GFP + Rapamycin or GFP + Rapa, E50K + Rapamycin or E50K + Rapa) treatment. **E**. Immunostaining of optineurin (OPTN), p62, PSMB5, and LC3 (in red) in GFP and E50K-GFP injected eyes without or with rapamycin treatment. Scale bar, 25 μm. **F**. Bar graph depicts the staining intensity (mean ± SEM, n ≥ 3) of OPTN, p62, PSMB5, and LC3 in retinal ganglion cell layer in each treatment group relative to that of the GFP control group. *, P < 0.015; #, P < 0.05 compared to GFP no treatment controls.

Rapamycin is an inhibitor of the Ser/Thr protein kinase named “mammalian target of rapamycin” (mTOR) [41]. Inhibition of mTOR mimics cellular starvation that induces autophagy by blocking signals required for cell growth and proliferation. Rapamycin is thus also recognized as an autophagic inducer. Rats injected with AAV2-GFP- and AAV2-E50K-GFP-vectors were treated with rapamycin to determine whether this treatment can reduce or rescue optineurin-mediated phenotypes in rats. Right after intravitreal injection of viral vectors, the treatment began by intraperitoneal injection of 1% v/w solution of rapamycin, 20 mg/kg body weight, as was successfully done previously in mouse studies [42]. The IOP, monitored at day 0, and 2, 4, and 5 weeks after viral injection, was not affected by rapamycin injections (Figure 6A). Results also indicated that following rapamycin treatments, the RGC (Figure 6B) and axonal (Figure 6C, right panel) counts in E50K-GFP-expressing eyes were increased, closer to the control level, compared to the E50K-without rapamycin treatment group. The RGC counts were 105105 ± 12750, 104323 ± 20113, 70633 ± 10543, and 82596 ± 11325, and the total axon counts were 739059 ± 12434, 80642 ± 16413, 540323 ± 46416, and 732678 ± 25307, respectively, for GFP, GFP + Rapamycin, E50K, and E50K + Rapamycin groups. The optic nerve axons of GFP control without and with rapamycin treatment groups displayed normal morphology. E50K-GFP-injected eyes showed a reasonably normal-appearing optic nerve cross section with rapamycin treatment, compared with the extensive degeneration without the treatment (Figure 6C, left panel). The extent of apoptosis (Figure 6D) was significantly (P = 0.0003) decreased. The apoptotic percentage was 1.4 ± 0.3, 1.7 ± 0.5, 11.7 ± 1.9, and 2.9 ± 0.3, respectively for GFP, GFP + Rapamycin, E50K, and E50K + Rapamycin groups.

Compared to GFP controls, the staining levels of optineurin and LC3 were significantly (p < 0.015) higher, and those of PSMB5 and p62 were lower, confirming that the proteasome function was compromised and autophagic process was activated in E50K transduced rat eyes. Rapamycin treatment in those rats caused a substantial reduction in the optineurin level (Figure 6E and F) while the LC3 level was further elevated (Figure 6E and F). In GFP viral vector injected specimens, the level of optineurin was similar with or without rapamycin administration (Figure 6E and F). Consistent with the rapamycin autophagy-inducing action, the LC3 staining was enhanced and the p62 staining was decreased in the GFP + Rapamycin group (Figure 6E and F). The level of PSMB5 was also diminished by rapamycin (Figure 6E and F), which has been reported previously to inhibit proteasome activity in rapamycin-fed mice [43].

## Discussion

Optineurin is a gene linked to NTG and ALS. It has previously been shown in cultured RGC5 cells that optineurin is ubiquitinated [32]. In normal homeostatic situation, the endogenous optineurin is processed mainly through the UPP pathway. Upon overexpression and/or mutation, the UPP function is inhibited and autophagy is induced for clearance of the protein [32].

The present study used AAV infection to extend the *in vitro* findings to *in vivo* adult rats. To date AAV has been the method of choice for gene delivery to RGCs [44-47]. Little or no signs of inflammation, cytotoxicity, abnormal growth, or immune reaction have been detected in the eyes following administration of AAVs [46]. There are many different serotypes of AAV, but AAV2 displays excellent tropism for RGCs, especially when injected into the vitreous [48]. Strong, constitutive promoters such as CMV and/or β-actin are commonly used to drive transgene expression [44,45,47].

We used pTR-SB-smCBA-V2 vector to construct AAV2-OPTN-GFP vectors. The advantage of using this plasmid is that the size of the smCBA promoter is smaller (approximately 1 kb) compared to the full-length CBA (about 1.7 kb), while it still exhibits an expression pattern similar to that of the full-length CBA in the retina [49]. Because of the smaller size, wild-type or E50K mutant OPTN-GFP fusion gene (about 2.5 kb) can be successfully packaged into viral particles.

In pilot experiments, moderate to strong GFP expression in RGCs was observed in rat eyes 5 weeks after a single intravitreal injection of AAV2-GFP containing a total of 5 × 10^10^ vp while little inflammation, cytotoxicity, or abnormal growth was noted. These parameters were thus selected for the study. The number of GFP-expressing green cells/field, the average intensity/cell, the total number of RGCs, and the integrity of optic nerve axons were compared between the AAV2-GFP injected eyes and non-injected or PBS-injected controls. As anticipated from reported observations [46], no significant difference was detected. By contrast, in wild-type and E50K optineurin-injected eyes, the retina was thinner, the RGC density was lower, the apoptosis level was higher, the axons were degenerated, and the axon counts were much reduced. These findings were consistent with the previous *in vitro* data, which showed that upregulated and mutated optineurin induced toxic effects such as apoptosis [50-53]. Also as *in vitro* [13,28,31,50,53], the deleterious optineurin phenotypes were more dramatically seen *in vivo* with the E50K mutation than the wild-type (Figures 1E, 2 and 4), suggesting that the observed consequences were, at least in part, related to the mutant, not merely a reflection of the overexpressed protein level. Of note in addition is that during the course of the experiment, the IOP in rats after viral delivery of the optineurin gene to RGCs, as expected from the clinical perspective, was not increased.

The previous *in vitro* findings were established using mostly RGC5 cells, an immortalized rat RGC cell line created originally by transforming postnatal day 1 rat retinal cells with E1A adenovirus [54,55]. These cells, while having been used extensively in the field, are now shown to be a different retinal cell type, namely, mouse SV-40 T antigen transformed photoreceptor 661 W cells [38]. It is thus imperative to validate the results in *in vivo* animal models. We herein demonstrated that in rat eyes, viral expression of wild-type and E50K optineurin in RGC layer did result in a declined PSMB5, an increased LC3, as well as a reduced p62 levels in the RGC layer, confirming that an impaired UPP function and induced autophagic process previously documented in RGC5 cultures ([32] and unpublished observations) also occurred *in vivo*.

Interestingly, in a chronic ocular hypertension rat model [39,40], a downregulation of the proteasomal activity in the RGC layer was also observed by PSMB5 immunostaining in eyes with optic nerve injuries (Figure 5). In this rat model, optineurin was in addition found increased and autophagy was activated in advanced injury (Figure 5). Optineurin expression has been shown to be upregulated with sustained pressure elevation in human perfusion organ cultures [56,57]. Autophagy has also been demonstrated to be activated in RGCs in other models after optic nerve transection and ischemia and with acute or chronic IOP elevation in rats [58-60].

Autophagy has emerged as a key process with implications in disease conditions such as cancer and neurodegenerative diseases. It is a major degradation pathway for aggregate-prone, intracytosolic proteins causing neurodegenerative disorders such as Huntington’s disease [61]. Upregulating autophagy has been shown to be a promising therapeutic intervention for clearing these disease-causing proteins [61]. Rapamycin, a compound already approved by the U.S. Food and Drug administration [62], and its analogs have been used previously to reduce huntingtin levels and attenuate the mutant huntingtin toxicity in cell, fly and mouse models of disease [42]. While rapamycin may also affect pathways other than autophagy [63,64], its protective effect was attributed, at least partially, to enhanced clearance of the mutant protein via autophagy. Rapamycin, lithium, and trehalose have also been shown to increase clearance of autophagy substrates α-synuclein and mutant huntingtin [65,66]. We applied a similar strategy and found that rapamycin was effective to reduce the overexpressed E50K optineurin, avert the optineurin phenotype, and increase cell survival in our *in vivo* rat model. Currently, investigators are actively searching for small and safe molecules to enhance autophagy [67,68]. Reduction of the optineurin level and rescue of RGCs from cell death by various approaches *in vivo*, if successful, will certainly have high translational impact.

## Conclusions

Optineurin is a gene linked to NTG and ALS. It has been shown previously in cultured RGC5 cells that optineurin is ubiquitinated. In normal homeostatic situation, the endogenous optineurin is processed mainly through the UPP pathway. Upon overexpression and/or mutation, the UPP function is compromised and autophagy is induced for clearance of the protein. We herein demonstrated that in rat eyes, viral expression of wild-type and E50K optineurin in RGC layer also resulted in an impaired UPP function and an induced autophagic process. The level of PSMB5 and p62 was declined and LC3 was elevated *in vivo*, confirming the previous RGC5 *in vitro* results. Apoptosis was in addition observed. Furthermore, rapamycin was found effective in reducing the overexpressed E50K optineurin, averting the optineurin phenotype, and increasing cell survival in the *in vivo* rat model. Reduction of the optineurin level and rescue of RGCs from cell death by rapamycin and other analogs may have high translational impact.

## Methods

### Construction of serotype 2 AAV vectors

AAV vector plasmid psmCBA-OPTN_WT_-EGFP or psmCBA-OPTN_E50K_-EGFP was constructed by inserting human OPTN_WT_-EGFP or OPTN_E50K_-EGFP fusion gene into empty vector pTR-SB-smCBA-V2 (generously provided by Dr. Sanford L. Boye of University of Florida) which contains a ubiquitous smCBA promoter (small CMVie enhancer and chicken β-actin hybrid promoter). Briefly, OPTN_WT_-EGFP or OPTN_E50K_-EGFP was digested with XhoI and NotI from plasmid pOPTN_WT_-EGFP or pOPTN_E50K_-EGFP and ligated into XhoI and NotI digested vector pTR-SB-smCBA-V2. Control vector plasmid psmCBA-EGFP was additionally made using a similar strategy. AAV2 vectors were packaged according to previously published methods [69] (packaging service was provided by Powell Gene Therapy Center of University of Florida). Viral particles were resuspended in phosphate buffered saline (PBS) (Sigma, St. Louis, MO, U.S.A.) and the titer was determined by dot blot assays. The resulting titers were 1.37 × 10^13^ vg/ml, 2.00 × 10^13^ vg/ml and 1.51 × 10^13^ vg/ml for AAV2-smCBA-OPTN_WT_-EGFP (AAV2-OPTN_WT_-GFP), AAV2-smCBA-OPTN_E50K_-EGFP (AAV2-OPTN_E50K_-GFP), and AAV2-smCBA-EGFP (AAV2-GFP) respectively.

### Animals

The study was carried out in adult male Brown Norway rats (170–200 g). All animal procedures were performed in compliance with the Association for Research in Vision and Ophthalmology (ARVO) statement for the Use of Animals in Ophthalmology and Vision Research. The Brown Norway rat has been a popular research animal used by various investigators in ophthalmology to create glaucoma or ocular hypertension models [9,46,70-72]. This animal is mild and not aggressive as other species and thus can be easily handled [40]. In addition, their relatively prominent globes and docile nature allow IOP measurement in the awake state [73]. A total of 78 animals were used.

### Intravitreal injection

The left eyes of rats were injected with 5 × 10^10^ vp (2.5 to 3.7 μl) of one of recombinant AAV2 vectors (AAV2-GFP, AAV2-OPTN_WT_-GFP or AAV2-OPTN_E50K_-GFP). Before intravitreal injection, the eyelids and conjunctival cul de sac were cleaned and rinsed with 0.5% betadine solution. Intravitreal injections into the vitreous chamber were performed with a Zeiss operating microscope under general anesthesia by intraperitoneal injection of standard rat cocktail consisting of ketamine (100 mg/Kg) and xylazine (5 mg/Kg), using a 10 μl Hamilton syringe (Hamilton Company, Reno, NV, U.S.A.) adapted to a customized pulled glass needle. The contralateral (right) eyes were injected with PBS, or left non-injected as controls. The sclera was exposed and the tip of the needle was inserted at a 45° angle through the sclera and retina into the vitreous space. The solution was injected slowly over a period of approximately 1 min [73]. Each dosage was split and injected into two different locations in the same eye to ensure even distribution of the virus in the eye. Care was taken to avoid injury to the lens [3,46]. After gentle withdrawal of the needle, erythromycin ophthalmic ointment was applied to the injection site. Post viral vector delivery, the rats were administrated one subcutaneous injection of buprenorphine (0.1 mg/Kg) to decrease eye rubbing. The rats were subjected to non-invasive weekly examination with a slit-lamp while anesthetized to monitor for accidental lens puncturing and signs of inflammation. The rats were also examined weekly with an indirect ophthalmoscope to detect retinal damage and detachment. Rats with punctured lens or damaged retina were excluded.

### Chronic ocular hypertension model

Hypertonic saline (NaCl) was injected into episcleral veins of one eye of Brown Norway rats to obstruct the aqueous outflow [39,40] and achieve chronic IOP elevation. The contralateral eyes were left non-injected as control. The optic nerve head and retinal vasculature were assessed [40]. Degenerating axons were characterized by axonal swellings and myelin debris [40]. The optic nerve damage in the rat models was graded from 1 (no injury) to 5 (active degeneration in the entire optic nerve area) [74].

### IOP measurements

The IOP was measured at day 0 (before injection), and weeks 2, 4 and 5 post-injection with rebound tonometer TonoLab (Icare Finland, Helsinki, Finland) in awake rats. Ten consecutive readings were taken per eye without any anesthesia. The highest and lowest values were discarded and the mean IOP was recorded as described previously [73]. The IOP in eyes of all treatment groups was compared.

### Transgene expression

Five weeks after intravitreal injection, animals were euthanized by CO_2_ inhalation. The eyes were enucleated and the anterior segments were removed. The retinal flat mount (or whole mount) was prepared for transgene expression. Briefly, the anterior segment was removed and the posterior segment was fixed in 4% paraformaldehyde for 30 min. The retina dissected was further fixed briefly, and carefully transferred to a microscope slide with the RGC layer side up. Relieving incisions were made to divide the retina into quadrants and to allow the retina to flatten. Under Zeiss confocal microscope (Carl Zeiss, Thornwood, NY, U.S.A.), the GFP green fluorescence was visualized in a masked manner. The number of GFP-positive cells was counted in digital images taken at 20x (0.125 mm^2^) under microscope from flat mounts in 20 standard fields in an equally spaced fashion from the center of the retina to the periphery in each quadrant [75]. The total number of green cells in these 20 fields was calculated [76]. Comparisons between the AAV2- and AAV2-OPTN_WT_- and AAV2-OPTN_E50K_-GFP-injected groups (n = 15) were made using ANOVA.

### Histochemical staining, immunofluorescence and apoptosis

The retinal flat mounts were further fixed overnight in 4% paraformaldehyde, and the retinas were processed and embedded in paraffin. Sections (6 μm) were used to visualize the GFP or optineurin-GFP fluorescence directly under a Zeiss fluorescence microscope (Carl Zeiss), in a masked fashion after AAV2-, AAV2-OPTN_WT_- and AAV2-OPTN_E50K_-GFP injections.

Paraffin sections were also obtained from Brown Norway rats with chronic IOP elevation [39,40]. For the first set of immunostaining experiments, sections from rat eyes (3 each) with optic nerve damage graded as 1, 2.75 (degenerating axons and axonal swelling spread more than the focal area), and 5 were used. The mean IOP readings in those eyes were 21.6, 27.2, and 37.2 mmHg, respectively. For the second immunostaining experiments, sections from rat eyes (n = 4 or 5 each) with 1, 2.6 to 3.0 (2.58, 2.75, 2.75, and 3), and 5 optic nerve injury grades were used.

The sections were deparaffinized and immunostained with polyclonal anti-optineurin (1:100, Cat. 100000, Cayman Chemical, Ann Arbor, MI, U.S.A.) and Cy3-labeled secondary antibody to yield positive staining products in red for transgene expression assessments. The morphology of the retina was examined by H and E staining. The number of RGCs was also counted.

Immunohistochemical staining for PSMB5 with anti-PSMB5 (1:100, Cat. Ab3330, Abcam, Cambridge, MA, U.S.A.), for p62 with anti-p62 (1:1000, Cat. PM045, MBL International, Woburn, MA, U.S.A.), and for LC3 with anti-LC3 (1:100, Cat. PD015, MBL International) was also carried out. Images were acquired using a 20 or 40× objective on an Axioscope (Carl Zeiss MicroImaging) with the aid of MetaMorph software (Molecular Devices, Downingtown, PA, U.S.A.). The staining intensity in the slides from the chronic ocular hypertension model was quantified by image analysis on selected images. The image file was converted from RGB to grey scale and Imaging Processing Tool kit 3.0 (Reindeer Games, Inc, Gainesville FL, U.S.A.) was used to measure the intensity of staining in at least 10 RGCs. Background intensity taken in empty spaces was subtracted. The staining intensity in RGCs from injury grade 2.6, 2.75, 3, and 5 slides was compared with that from grade 1 using Student’s t tests. Values of P < 0.05 were considered to be significant.

To examine apoptosis, TUNEL assay using an ApopTag kit from Millipore (Billerica, MA, U.S.A.) was performed. The percentage of apoptotic cells was calculated (from counts of total and apoptotic cells) in RGC layer on each specimen from 20 images of 10x fields.

### Western blot analysis

Five weeks following injections as described above, rat eyes were enucleated and placed in PBS. After removing the anterior portion of the eye and the lens, the retina was dissected from the eyecup disc and dissolved in electrophoresis sample buffer, boiled and subjected to Western blotting for optineurin, PSMB5 and LC3 levels [32].

### Axon counts

The optic nerves in the enucleated eyes were fixed with 2% paraformaldehyde and 2.5% glutaraldehyde in 0.1 M sodium cacodylate buffer, pH 7.4, post fixed in 1% osmium tetroxide for 2 h and in 0.25% uranyl acetate for an additional 2 h. The nerves were dehydrated with series of alcohol and propylene oxide, and embedded in Embed-812 medium (Electron Microscopy Sciences, Hatfield, PA, U.S.A.). The axons in the optic nerve in rat eyes (n = 15/treatment group) either injected with PBS, AAV2-GFP, AAV2-OPTN_WT_-GFP, or AAV2-OPTN_E50K_-GFP were evaluated as described by Quigley and co-workers [75]. Briefly, semi-thin (0.9 μm) sections were stained with 1% toluidine blue and imaged with spinning disk live-cell imaging system (Carl Zeiss) with 10x (for measurement of area of optic nerve) or 100x (for estimation of axon count) objective. The dark staining myelin was identified and the myelinated fibers were enumerated [77,78]. The density of axons in high-power fields in 10 selected, non-overlapping areas in central, superior, inferior, temporal, and nasal optic nerve, were determined by MetaMorph. The total nerve fiber count was estimated by multiplying density with the area to yield axon count [70,77]. Counts from all treatment groups were compared. Statistical differences were evaluated by ANOVA.

### Effects of rapamycin

The eyes of two sets of rats were injected with either AAV2-GFP (n = 4) or AAV2-OPTN_E50K_-GFP (n = 10) as above. Half of each set of animals received rapamycin (20 mg/Kg body weight) (Sirolimus, LC Lab, Woburn, MA, U.S.A.) via intraperitoneal injection [42,79] 3 times a week for 5 weeks. The rats were subsequently sacrificed, and the eyes were enucleated for flat mount, light microscopy, RGC counts, Western blotting, immunostaining for optineurin, p62, PSMB5, LC3, and TUNEL staining, and total nerve fiber count as described above.

## Abbreviations

AAV2: Adeno-association type 2 virus
ALS: Amyotrophic lateral sclerosis
ARVO: The Association for Research in Vision and Ophthalmology
GFP: Green fluorescence protein
IOP: Intraocular pressure
LC3: Microtubule-associated protein 1A/1B-light chain 3
NTG: Normal tension glaucoma
OPTN: Optineurin
PBS: Phosphate buffered saline
POAG: Primary open angle glaucoma
PSMB5: Proteasome β5 subunit
RGC: Retinal ganglion cell
UPP: Ubiquitin-proteasome pathway
vp: Viral particles
